# Patients’ Perceptions Regarding Acceptance of Dental Implants as an Option for the Replacement of Missing Teeth: An Observational Study

**DOI:** 10.7759/cureus.57232

**Published:** 2024-03-30

**Authors:** Sharanamma B Bhagawati, Saurabh R Jain, Puja Debnath, Khadeer Riyaz, Rohit Patil, Jaweria Ansari

**Affiliations:** 1 Department of Periodontics, Hazaribagh College of Dental Sciences and Hospital, Hazaribagh, IND; 2 Department of Prosthodontics, Jawahar Medical Foundation’s Annasaheb Chudaman Patil Memorial Dental College, Dhule, IND; 3 Department of Periodontics, Agartala Government Dental College & IGM Hospital, Agartala, IND; 4 Department of Orthodontics, The Oxford Dental College and Research Centre, Bengaluru, IND

**Keywords:** observational study, replacement, perception, tooth loss, dental implants

## Abstract

Introduction: Dental implants enhance the self-assurance and overall well-being of individuals by providing oral comfort during mastication and a notable degree of contentment. The objectives of the present study were to assess patients’ perception of opting or non-opting for dental implants as a replacement for missing teeth and to determine the correlation between various factors and perceived demand for dental implant treatment.

Materials and methods: A cross-sectional observational study was conducted on 214 partially edentulous individuals aged between 21 and 50 years. These patients sought treatment to replace their missing teeth. The participants were provided with detailed information regarding various options for replacing their missing teeth, including removable prostheses, fixed partial dentures, and dental implants. The researchers recorded and evaluated the reasons behind the patients' decision to opt for or decline dental implant treatment using the chi-squared test. Categorical variables were summarized as percentages (n %). The association between variables and binary data was examined using point biserial correlation, whereas, for continuous data, the Pearson correlation coefficient was employed.

Results: About 65 (30.4%) patients opted for dental implant treatment and 149 (69.6%) patients did not opt for dental implant treatment. Missing teeth were found in 120 women (56.08%) and 94 men (43.92%). The main reason for seeking dental implant treatment was the need for improvement in functions such as chewing in 65 (100%) patients, followed by the need for improvement in oral health in 57 (88%), aesthetics in 54 (83%), need for bone and adjacent teeth preservation in 52 (80%), and durability of dental implants in 46 (71%) patients. The main reasons for not seeking dental implant treatment and opting for fixed prostheses other than dental implants or removable prostheses cost 149 (100%), fear of surgery 132 (91%), underlying health issues 121 (81%), lack of knowledge about dental implants 120 (80.5%), and time management issues 92 (62%). Gender, age, and number of missing teeth showed a negative correlation, whereas level of education, social status, and oral health awareness showed a positive correlation with the perceived need for dental implant treatment.

Conclusion: Dental implant treatment was preferred by 30.4% of patients, which was influenced by gender, sex, level of education, social status, awareness of oral health, and number of missing teeth. Cost, fear of surgery, underlying medical conditions, lack of knowledge, and time management are some reasons for not opting for dental implant treatment.

## Introduction

Tooth loss continues to be a prominent obstacle to oral well-being, and has a negative impact on individuals' dietary consumption and nutritional well-being, thereby compromising their overall health. In India, it has been discovered that 10.7% of the population experiences complete tooth mortality, while 58.8% experience partial tooth mortality. Moreover, this rate is higher in rural populations than in urban populations [1].

Restoration of missing teeth has been a primary objective of dental professionals throughout history, aiming to recover the patient's functionality, speech, aesthetics, and psychological well-being. Currently, diverse alternatives are available to address this issue, such as complete and partial removable prostheses, fixed prostheses other than dental implants (FPODI), dental implants, and overdentures [2]. Dental implants have demonstrated significant longevity and enhanced functionality compared with FPODI and removable dentures. Numerous studies have provided evidence that dental implants substantially enhance mechanical aspects such as retention, stability, and support of dentures, leading to functional contentment and an improvement in the overall quality of life [3-5].

The success of dental implants is based on patients’ perceptions and attitudes toward dental implant treatment as well as their knowledge about the treatment [2]. Despite numerous advantages and promotion of dental implants as the preferred treatment, their use remains limited. Studies have indicated that patients' inclination towards accepting implant therapy is primarily influenced by the financial burden associated with the treatment, intricate surgical and prosthetic procedures involved, adverse health conditions, and a lack of awareness [4]. Limited knowledge is available on various factors affecting patients’ perception of dental implant treatment and reasons for opting or not opting for such treatment. Understanding the patients' perspective regarding dental implants is beneficial as it allows for aligning their expectations with realistic outcomes. This helps to prevent the formation of a negative perception towards implants, which can be caused by a lack of effective communication by dentists and dissatisfaction among consumers. The present investigation was undertaken to provide data concerning the perception of a cohort of Indian individuals towards dental implants as a potential solution for the replacement of missing teeth and reasons for opting or not opting for dental implant treatment. This study also aimed to establish the correlation between different variables and patient perceptions.

## Materials and methods

Study design

This observational study was conducted in the outpatient department (OPD) of the Department of Periodontics and Prosthodontics, Annasaheb Chudaman Patil Memorial Dental College, Dhule, Maharashtra, from December 2022 to December 2023. Ethical approval was obtained from the JMF ACPM Dental College Institutional Ethics Committee (EC/NEW/INST/2022/2959/054). Written informed consent was obtained from all the participants who agreed to participate in the study. No incentives were provided to the patients for their participation.

Sample size calculation

Convenience sampling was used in this study. The sample size was calculated using G Power software (version 3.2.9, G Power, Aichach, Germany). Power analysis revealed that 214 samples provided a power of 95%, with a type 1 error of 5% as the population proportion of 17% was used according to previous studies [2].

Eligibility criteria and methodology

This study included 214 participants (94 males and 120 females) who fulfilled the eligibility criteria. Only new partially edentulous patients aged 21-50 years, who reported to the departments during the study period and agreed to replace their missing teeth were included in the study. Patients who had received dental implant treatment before, those with severe bone loss, and those who were completely edentulous, and unwilling to participate were excluded from the study.

To ensure consistency, all patients were assessed by postgraduate students supervised by an experienced faculty member. Each student evaluated the patient's oral cavity, completed a patient examination document, and presented all the collected information to the supervisor. This document included detailed demographic data, including age, sex, socioeconomic status, educational level, number of dental visits within the past year, frequency of tooth brushing to evaluate attitude and awareness of oral health, and the number of missing teeth. Subsequently, comprehensive information was provided to all patients regarding the available alternatives for replacing missing teeth, such as FPODI, removable prostheses, and dental implant treatments, along with their respective advantages and disadvantages. Patients were then asked to indicate their reasons for choosing or not choosing dental implant treatment (Table 1). Each participant was allowed to select one or more options.

**Table 1:** Factors for opting or not opting for dental implant treatment

Factors for opting for dental implants	
1	Improved aesthetics
2	Improved function
3	Durability of dental implants
4	Bone/teeth preservation
5	Improved oral health
Factors for not opting for dental implants	
1	Lack of knowledge about dental implants
2	Cost of dental implants
3	Fear from surgery
4	Time commitments for dental visits
5	Health issues

Statistical analysis

Statistical analysis was performed at a 95% confidence level with a statistical significance of p-value less than 0.05, using the IBM SPSS Statistics for Windows, Version 21 (Released 2012; IBM Corp., Armonk, New York, United States). Categorical variables were summarized as percentages. Categorical data were analyzed using the chi-square test. The correlation between variables with binary data such as sex, social status, level of education, oral health awareness, and perceived demand for dental implants was analyzed using point biserial correlation. The correlation between variables and continuous data, such as gender, number of missing teeth, and perceived demand for dental implants, was analyzed using the Pearson correlation coefficient.

## Results

**Figure 1:**
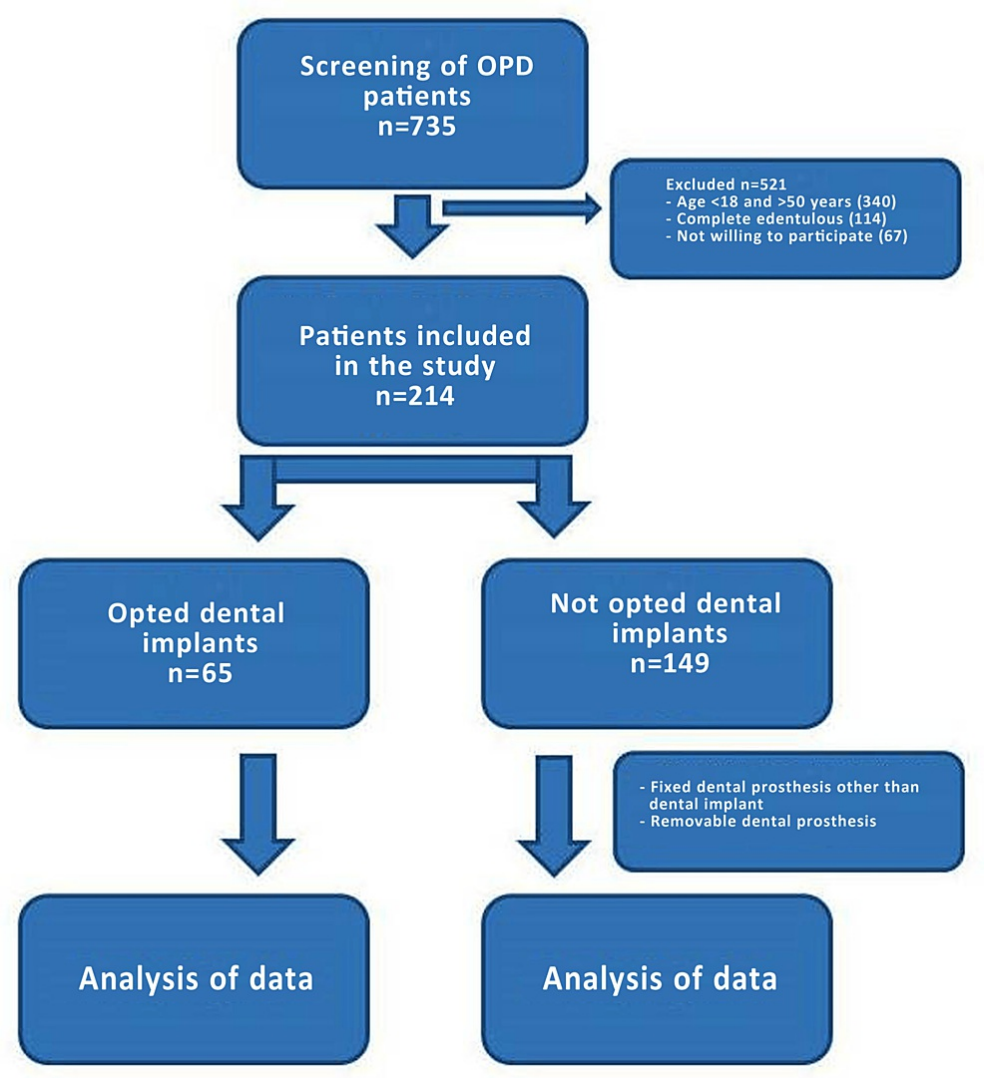
Study design

A total of 735 patients who reported OPD in the Department of Periodontics and Prosthodontics were screened, and 214 who fulfilled the eligibility criteria were included in the study. About 65 patients (30.4%) opted for dental implant treatment, and 149 patients (69.6%) did not opt for dental implant treatment, as shown in Figure 1.

No statistically significant gender difference was observed for patients who opted or did not opt for dental implant treatment (p=0.302); however, a greater number of females (58.38%) did not opt for dental implants. Non-significant differences were found between the different age groups (p=0.366); however, most patients (47.20%) with missing teeth were in the age group 31 to 40 years. Forty percent of the participants who opted for dental implants were also from the same age group. About 35.51% of patients with one or two missing teeth wanted to replace their missing teeth, and a dental implant was chosen as a replacement option by 44.62%, and 31.54% of patients chose FPODI or removable prostheses as replacement options, and this difference was statistically significant (p=0.001). Furthermore, dental implants are mainly required to replace one or two missing teeth, whereas FPODI or removable prostheses are required to replace more than three missing teeth. A total of 103 (48.13%) patients who received higher education wanted to replace their missing teeth, 51 (78.46%) opted for dental implants, and 52 (34.89%) opted for FPODI or removable prostheses. The demand for dental implant treatment was greater in the urban population (69.23%) than in the rural population (30.77%). Regarding oral health awareness, 125 (58.41%) patients used the incorrect brushing technique, 117 (54.67%) brushed only once a day, 159 (74.30%) did not use floss, 168 (78.50%) did not use an interdental brush, and 101 (47.20%) visited the dentist only once in the previous year for oral health check-ups. About 64 (42.95%) patients opted for FPODI and 85 (57.05%) opted for removable prostheses (Table 2).

**Table 2:** Demographic details of the study participants *p-value<0.05: Significant; NS: Non-significant

Variables	Dental implants n (%)			p-value
	Not opted 149 (69.6%)	Opted 65 (30.4%)	Total 214 (100%)	
Gender n (%)				
Female	87 (58.38%)	33 (50.76%)	120 (56.08%)	0.302 (NS)
Male	62 (41.62%)	32 (49.24%)	94 (43.92%)	
Age group (years) n (%)				
21-30	38 (25.50%)	21 (32.31%)	59 (27.57%)	0.366 (NS)
31-40	75 (50.34%)	26 (40.00%)	101 (47.20%)	
41-50	36 (24.16%)	18 (27.69%)	54 (25.23%)	
Number of missing teeth n (%)				
1-2	47 (31.54%)	29 (44.62%)	76 (35.51%)	0.001*
3-5	38 (25.50%)	9 (13.85%)	47 (21.96%)	
6-8	43 (28.86%)	8 (12.31%)	51 (23.83%)	
>8	21 (14.09%)	19 (29.23%)	40 (18.69%)	
Level of education n (%)				
Higher	52 (34.89%)	51 (78.46%)	103 (48.13%)	0.001*
Primary	47 (31.54%)	0 (0.00%)	47 (21.96%)	
Secondary	50 (33.57%)	14 (21.54%)	64 (29.91%)	
Social status n (%)				
Rural	86 (57.72%)	20 (30.77%)	106 (49.53%)	0.001*
Urban	63 (42.28%)	45 (69.23%)	108 (50.47%)	
Brushing technique n (%)				
Correct	58 (38.93%)	31 (47.69%)	89 (41.59%)	0.231 (NS)
Incorrect	91 (61.07%)	34 (52.31%)	125 (58.41%)	
Brushing frequency n (%)				
Once	91 (61.07%)	26 (40.00%)	117 (54.67%)	0.004*
Twice	58 (38.93%)	39 (60.00%)	97 (45.33%)	
Flossing habit n (%)				
No	119 (79.87%)	40 (61.54%)	159 (74.30%)	0.005*
Yes	30 (20.13%)	25 (38.46%)	55 (25.70%)	
Interdental brush n (%)				
No	128 (85.91%)	40 (61.54%)	168 (78.50%)	0.001*
Yes	21 (14.09%)	25 (38.46%)	46 (21.50%)	
Number of dental visits in last one year n (%)				
0	65 (43.62%)	14 (21.54%)	79 (36.92%)	0.002*
1	59 (39.60%)	42 (64.62%)	101 (47.20%)	
2	25 (16.78%)	9 (13.84%)	34 (15.89%)	
Type of prosthesis demanded n (%)				
Fixed	64 (29.90%)	65 (100.00%)	129 (60.28%)	0.001*
Removable	85 (39.72%)	0 (0.00%)	85 (39.72%)	

Perceived reasons for demanding dental implant 

The main reason for seeking dental implant treatment was the need for improvement in functions such as chewing (65 patients, 100%), followed by the need for improvement in oral health (57 patients, 88%), aesthetics (54 patients, 83%), need for bone and adjacent teeth preservation (52 patients, 80%), and durability of dental implants (46 patients, 71%). The demand for dental implants to improve oral function was observed in both males (32 males, 49.23%) and females (33 females, 50.77%). Patients who received higher education showed greater demand for dental implant treatment for improvement in function (51 patients, 78.46%), oral health (46 patients, 80.70%), aesthetics (42 patients, 77.78%), and preservation of bone and adjacent teeth (41 patients, 78.85%). Patients from the urban population and those with good oral health awareness showed an inclination toward dental implants. The demand for dental implants was greater in patients aged 31-40 years mainly for improvement in oral function (26 patients, 40.00%) and aesthetics (23 patients, 42.59%). Furthermore, the replacement of one or two posterior teeth was mainly required for improvement in oral function (29 patients, 44.62%) and oral health (25 patients, 43.86%), whereas the replacement of one or two anterior teeth was mainly required to improve aesthetics (23 patients, 42.59%) and preserve bone and adjacent teeth (23 patients, 44.23%) (Table 3).

**Table 3:** Frequency distribution of factors for patients who opted for dental implant treatment n: Number of participants; %: Frequency

Variables	Aesthetics 54 (83%)	Function 65 (100%)	Durability 46 (71%)	Bone/teeth preservation 52 (80%)	Oral health 57 (88%)
Gender n (%)					
Male	26 (48.15%)	32 (49.23%)	19 (41.30%)	25 (48.08%)	28 (49.12%)
Female	28 (51.85%)	33 (50.77%)	27 (58.70%)	27 (51.92%)	29 (50.88%)
Level of education n (%)					
Primary	0 (0.00%)	0 (0.00%)	0 (0.00%)	0 (0.00%)	0 (0.00%)
Secondary	12 (22.22%)	14 (21.54%)	9 (19.57%)	11 (21.15%)	11 (19.30%)
Higher	42 (77.78%)	51 (78.46%)	37 980.43%)	41 (78.85%)	46 (80.70%)
Social status n (%)					
Urban	37 (68.52%)	45 (69.23%)	32 (69.57%)	35 (67.31%)	39 (68.42%)
Rural	17 (31.48%)	20 (30.77%)	14 (30.43%)	17 (32.69%)	18 (31.58%)
Oral health awareness n (%)					
Good	32 (59.26%)	38 (58.46%)	27 (58.70%)	25 (48.08%)	32 (56.14%)
Poor	22 (40.64%)	27 (41.54%)	19 (41.30%)	21 (40.38%)	25 (43.86%)
Age group in years n (%)					
21-30	21 (38.89%)	21 (32.31%)	13 (28.26%)	21 (40.38%)	18 (31.58%)
31-40	23 (42.59%)	26 (40.00%)	22 (47.83%)	22 (42.31%)	21 (36.84%)
41-50	10 (18.52%)	18 (27.69%)	11 (23.91%)	9 (17.31%)	18 (31.58%)
Number of missing teeth n (%)					
1-2	23 (42.59%)	29 (44.62%)	19 (41.30%)	23 (44.23%)	25 (43.86%)
3-5	6 (11.11%)	9 (13.85%)	4 (8.70%)	5 (9.62%)	9 (15.79%)
6-8	6 (11.11%)	8 (12.31%)	8 (17.39%)	6 (11.54%)	7 (12.28%)
>8	19 (35.19%)	19 (29.23%)	15 (32.61%)	18 (34.62%)	16 (28.07%)

Perceived reasons for not demanding dental implants

The main reasons for not seeking dental implant treatment and opting for FPODI or removable prostheses were cost (149 patients, 100%), followed by fear of surgery (132 patients, 91%), underlying health issues such as uncontrolled diabetes, hypertension, kidney, liver, or cardiovascular problems, chronic smokers (121 patients, 81%), lack of knowledge about dental implants with apprehension about choosing a new modality that was previously unknown to them (120 patients, 80.5%), and time management associated with a greater number of dental visits for dental implants (92 patients, 62%). The non-demand for dental implants was noticed mainly for cost issues for both males (62 males, 41.61%) and females (87 females, 58.39%). Patients who received higher education showed greater non-demand for dental implant treatment because of the cost (52 patients, 34.90%), fear of surgery (46 patients, 34.85%), and lack of knowledge (45 patients, 37.50%). Patients from the rural population, with poor oral health awareness, aged 31 to 40 years, and with one or two missing teeth, showed rejection of dental implant treatment (Table 4).

**Table 4:** Frequency distribution of factors for patients who did not opt for dental implant treatment n: Number of participants; %: Frequency

Variables	Time management 92 (62%)	Lack of knowledge 120 (80.5%)	Fear of surgery 132 (91%)	Health issues 121 (81%)	Cost 149 (100%)
Gender n (%)					
Male	38 (41.30%)	50 (41.67%	57 (43.18%)	49 (40.50%)	62 (41.61%)
Female	54 (58.70%)	70 (58.33%)	75 (56.82%)	72 (59.50%)	87 (58.39%)
Level of education n (%)					
Primary	36 (39.13%)	37 (30.83%)	42 (31.82%)	43 (35.54%)	47 (31.54%)
Secondary	30 (32.61%)	38 (31.67%)	44 (33.33%)	42 (34.71%)	50 (33.56%)
Higher	26 (28.26%)	45 (37.50%)	46 (34.85%)	36 (29.75%)	52 (34.90%)
Social status n (%)					
Urban	34 (36.96%)	51 (42.50%)	56 (42.42%)	42 (34.71%)	63 (42.28%)
Rural	58 (63.04%)	69 (57.50%)	76 (57.58%)	79 (65.29%)	86 (57.72%)
Oral health awareness n (%)					
Good	10 (10.87%)	30 (25.00%)	18 (13.64%)	14 (11.57%)	32 (14.77%)
Poor	82 (89.13%)	90 (75.00%)	114 (86.36%)	107 (88.43%)	117 (85.23%)
Age group in years n (%)					
21-30	26 (28.26%)	29 (24.17%)	33 (25.00%)	33 (27.27%)	38 (25.50%)
31-40	52 (56.52%)	61 (50.83%)	66 (50.00%)	66 (54.55%)	75 (50.34%)
41-50	14 (15.22%)	30 (25.00%)	33 (25.00%)	22 (18.18%)	36 (24.16%)
Number of missing teeth n (%)					
1-2	27 (29.35%)	37 (30.83%)	39 (29.55%)	36 (29.75%)	47 (31.54%)
3-5	25 (27.17%)	31 (25.83%)	36 (27.27%)	36 (29.75%)	38 (25.50%)
6-8	25 (27.17%)	33 (27.50%)	39 (29.55%)	31 (25.62%)	43 (28.86%)
>8	15 (16.30%)	19 (15.83%)	18 (13.64%	18 (14.88%)	21 (14.09%)

There was a weak negative correlation between gender and perceived demand for dental implants (r=-0.23), which was not statistically significant (p>0.05). This showed that females showed a greater inclination towards dental implant treatment than males. There was a very weak negative correlation between age and perceived demand for dental implants (r=-0.15), which was statistically significant (p<0.05). This showed that younger individuals demanded more dental implant treatment than older individuals. The number of missing teeth requiring replacement showed a moderate negative correlation with the perceived demand for dental implants (r=-0.43), which was statistically significant (p<0.05). This showed that dental implants were chosen to replace a smaller number of teeth (one or two), whereas FPD or removable prostheses were chosen to replace a greater number of teeth (more than three). The level of education and social status showed a weak positive correlation with perceived demand for dental implants (r-value of 0.27 and 0.01, respectively), which was statistically non-significant (p>0.05). This indicates that individuals with higher education and urban populations demand more dental implants. Awareness of oral health showed a strong positive correlation with perceived demand for dental implants (r=0.65), which was statistically significant (p-value<0.05). This indicated that as awareness of oral health increased, the demand for dental implant treatment also increased (Table 5).

**Table 5:** Correlation between various variables and factors for opting for dental implant treatment *Point biserial correlation; **Pearson correlation; ***p<0.05 significant; NS: Non-significant r-value of 0-0.199: very weak correlation; 0.2-0.399: weak correlation; 0.4-0.599: moderate correlation, 0.6-0.799: strong correlation

Variables	r-value	p-value
Gender*	-0.23	0.677 (NS)
Age**	-0.15	0.034***
Number of missing teeth**	-0.43	0.002 ***
Level of education*	0.27	0.561 (NS)
Social status*	0.01	0.891 (NS)
Oral health awareness*	0.65	0.039 ***

## Discussion

The present study aimed to assess the patient’s perception of opting for dental implant treatment and to find correlations between various factors such as age, gender, oral health awareness, socioeconomic status, level of education, number of missing teeth, and their perception of dental implant treatment.

Only 30.4% of the patients chose dental implant treatment, 29.90% chose FPODI, and 39.7% chose removable prostheses. Most patients opted for removable prostheses rather than implants owing to financial constraints. This is in agreement with the previous studies by Yakar et al. [5], Saha et al. [6], and Gupta et al. [3]. This finding contradicts the results reported by Siddique et al. [7] and Mayya et al. [2], who observed that the majority of participants (84.4% and 78.1%, respectively) recognized the perception of dental implants as an essential requirement. The disparity in findings can be attributed to variations in the educational background and awareness levels of patients.

The need for the replacement of missing teeth was noticed more in females, individuals with good oral health awareness, those who received higher education, and those aged 31-40 years. This was in accordance with previous studies [1,3,5]. Age and dental implant consideration had a negative correlation, indicating that the younger population was more eager for implant placement than older individuals who had a fear of surgery, underlying medical conditions, more tolerance for complete dentures, and higher cost. Most countries, including India, do not provide medical insurance for dental implant placements. This finding was in agreement with previous studies [5,8]. The level of education showed a positive correlation with dental implant consideration, indicating that patients with higher education could opt for dental implant treatment more than those with a low level of education. This finding was in agreement with previous studies [5,9]. However, individuals with a higher level of education and lower financial income may not choose to undergo dental implant treatment, as was observed in the present study. Consequently, the decision to opt for dental implants is influenced by both the patient's educational background and financial situation.

Cost was the main reason for not opting for an implant treatment. This could be because most patients who reported to dental colleges for treatment had low incomes, as noted in previous studies conducted on the Indian population [2,6,7]. Our finding was in contrast to that of Simensen et al., who reported that cost was not the deciding factor [10]. This could be attributed to the fact that the study was carried out on individuals who were referred from implant clinics, thus already exhibiting a high level of motivation towards dental implant treatment. However, it has been reported in many non-native studies that over a long-term horizon of 10-20 years, dental implant treatment is more cost-effective than FPODI [11,12].

A lack of information has also been identified as a primary factor in the current investigation of declining dental implant therapy. Most patients, especially those from rural areas, are unfamiliar with dental implants. After providing patients with information about dental implants, their perception of dental implant treatment was altered, and they exhibited a favorable attitude toward the treatment. Similar findings have been reported previously [4,6]. Most patients fear dental implant failure and surgical procedures associated with implant placement. Therefore, they were apprehensive about choosing a dental implant treatment. Describing the occurrence of implant failure is a relatively straightforward task compared with describing the achievement or longevity of implants. This is due to the multitude of potential factors that can contribute to implant failure, including the clinician's level of expertise, the patient's overall health condition, any parafunctional habits they may have, and their smoking status [13].

The findings of the present study have revealed that patients were keen to choose dental implant therapy because of the desire to safeguard both the bone and neighboring teeth, as opposed to opting for FPODI. This is in agreement with the findings of Jain et al. [14]. The underlying medical condition of the patient, such as uncontrolled systemic conditions, poses an absolute contraindication, whereas patients on bisphosphonates, corticosteroids, immunosuppressants, hormonal therapy, and irradiation of the head and neck are at increased risk of osteoporosis and dental implant failure [15].

The current investigation demonstrated that the primary factor influencing the decision to undergo dental implant treatment for replacing posterior teeth is the desire to enhance oral function, specifically in relation to chewing. Conversely, for anterior teeth, the primary motivation for opting for dental implants is the need for improved aesthetics. This finding is supported by those of previous studies [2,6,7,16]. It was also noticed that the demand for dental implants was higher in the case of replacement of one or two teeth, whereas FPODI or removable prostheses were demanded more in cases of multiple missing teeth (more than three). The reason for choosing a dental implant over an FPODI for single-tooth replacement might be the preservation of adjacent teeth and improved bone quality with implants, as noted by Hebel et al. [17] and Jain et al. [14]. Moreover, most of our patients were from rural and low-income groups; therefore, increased cost and fear of surgery were the main reasons for not choosing dental implants for the replacement of multiple teeth.

The strength of the present study was the in-depth understanding of various reasons for choosing or not choosing dental implant treatment by relating them to various demographic factors, which is not possible in questionnaire studies with limited options and analysis. Moreover, our study also examined the correlation between various variables and the perceived demand for dental implant treatment.

Limitations

The primary constraint of the investigation was the non-inclusion of completely edentulous patients. The ongoing investigation focused on individuals ranging from 21 to 50 years of age, whereas completely edentulous individuals typically manifest at an age exceeding 55 years. The evaluation forms were not completed by a solitary researcher, but rather by a number of students, thus hindering the achievement of standardization. However, to reduce this bias, the forms were filled out only by third-year postgraduate students under the supervision of experienced staff members.

## Conclusions

The findings of the current investigation demonstrated that 30.4% of patients chose dental implants as a means to replace one or two missing teeth. The factors inhibiting individuals from selecting dental implant treatment included cost, apprehension regarding the surgical procedure, preexisting medical conditions, inadequate understanding, and difficulties in managing time. Conversely, the rationale for opting for dental implants encompasses the desire to enhance oral function, oral health, aesthetic appearance, and the preservation of the surrounding bone and adjacent teeth. It is imperative to raise awareness of dental implants through diverse public awareness campaigns and the establishment of counseling centers within the OPDs of private dental clinics and dental colleges. The public sector must exert special efforts to reduce the cost of implants to make them accessible to all individuals and enhance knowledge among those with less education.
